# Long-term safety and influence on growth in patients receiving sirolimus: a pooled analysis

**DOI:** 10.1186/s13023-024-03243-5

**Published:** 2024-08-15

**Authors:** Yang-Yang Wang, Li-Ping Zou, Kai-Feng Xu, Wen-Shuai Xu, Meng-Na Zhang, Qian Lu, Xin-Lun Tian, Ling-Yu Pang, Wen He, Qiu-Hong Wang, Yang Gao, Li-Ying Liu, Xiao-Qiao Chen, Shu-Fang Ma, Hui-Min Chen, Shuo Dun, Xiao-Yan Yang, Xiao-Mei Luo, Lu-Lu Huang, Yu-Fen Li

**Affiliations:** 1 Department of Pediatrics, The First Medical Center of PLA General Hospital, Chinese PLA General Hospital, Beijing, 100853 China; 2 Department of Pulmonary and Critical Care Medicine, Peking Union Medical College Hospital, Chinese Academy of Medical Sciences, Peking Union Medical College, Beijing, 100730 China; 3 Center of Epilepsy, Beijing Institute for Brain Disorders, Beijing, 100069 China; 4 Department of Pediatrics, Shandong Linyi People’s Hospital, Shandong, China; 5 Department of Neurology, Hebei Children’s Hospital, Hebei, China; 6 Department of Pediatrics, Binzhou Medical University Hospital, Shandong, China; 7 Deyang People’s Hospital, Sichuan, China; 8 Department of Pediatrics, The 904th Hospital of Joint Logistic Support Force, Jiangsu, China; 9 Department of Pediatrics, Zhujiang Hospital of Southern Medical University, Guangdong, China

**Keywords:** Sirolimus, Tuberous sclerosis, Safety, Adverse events, Physical growth

## Abstract

**Background:**

Sirolimus is increasingly utilized in treating diseases associated with mTOR pathway overactivation. Despite its potential, the lack of evidence regarding its long-term safety across all age groups, particularly in pediatric patients, has limited its further application. This study aims to assess the long-term safety of sirolimus, with a specific focus on its impact on growth patterns in pediatric patients.

**Methods:**

This pooled analysis inlcudes two prospective cohort studies spanning 10 years, including 1,738 participants (aged 5 days to 69 years) diagnosed with tuberous sclerosis and/or lymphangioleiomyomatosis. All participants were mTOR inhibitor-naive and received 1 mg/m²/day of sirolimus, with dose adjustments during a two-week titration period to maintain trough blood concentrations between 5 and 10 ng/ml (maximum dose 2 mg). Indicators of physical growth, hematopoietic, liver, renal function, and blood lipid levels were all primary outcomes and were analyzed. The adverse events and related management were also recorded.

**Results:**

Sirolimus administration did not lead to deviations from normal growth ranges, but higher doses exhibited a positive association with Z-scores exceeding 2 SD in height, weight, and BMI. Transient elevations in red blood cell and white blood cell counts, along with hyperlipidemia, were primarily observed within the first year of treatment. Other measured parameters remained largely unchanged, displaying only weak correlations with drug use. Stomatitis is the most common adverse event (920/1738, 52.9%). In adult females, menstrual disorders were observed in 48.5% (112/217).

**Conclusions:**

Sirolimus’s long-term administration is not associated with adverse effects on children’s physical growth pattern, nor significant alterations in hematopoietic, liver, renal function, or lipid levels. A potential dose-dependent influence on growth merits further exploration.

**Trial registration:**

Pediatric patients: Chinese clinical trial registry, No. ChiCTR-OOB-15,006,535. Adult patients: ClinicalTrials, No. NCT03193892.

**Supplementary Information:**

The online version contains supplementary material available at 10.1186/s13023-024-03243-5.

## Introduction

Sirolimus, also known as rapamycin, is an inhibitor of mechanistic (mammalian) target of rapamycin complex 1 (mTORC1). While initially used as an antirejection medication, its off-label application for diseases linked to mTOR overactivation has been reported to yield minimal side effects and enhanced efficacy in the past decade [1, 2]. Recent research even hails it as a potential avenue to decelerate age-related pathologies, rejuvenate the immune system, and extend lifespan [3–6]. Concerns about its long-term safety persist, though. The adverse events (AEs) most frequently reported include hyperlipidemia, recurrent infections, stomatitis, and ovarian toxicity [7].

Intriguingly, the safety profile of sirolimus exhibits considerable variability. Some trials have reported fewer AEs in experimental groups compared to controls [8, 9], while others highlight its low tolerability with patients discontinuing medication prematurely [10, 11]. To date, fewer studies have conclusively identified whether patient demographics, therapeutic regimens, or other factors underpin these discrepancies. Also, the impact of long-term mTOR inhibition on the physical growth of pediatric patients remains an unresolved question.

Studying the long-term safety of sirolimus presents several challenges. Recruiting suitable participants and designing appropriate control groups are particularly difficult. Transplant recipients and patients with malignancies, due to their complex medication profiles and health conditions, may not be ideal candidates for safety-focused studies due to the potential for overestimation of side effects. Moreover, while randomized controlled trials (RCTs) are gold standards for evaluating short-term safety, their typical duration is insufficient for observing long-term effects. For example, an RCT study provided evidence that patients receiving placebo had a higher incidence of severe pulmonary or upper respiratory AEs than those receiving sirolimus [9]. In the absence of control groups, these events might have been erroneously attributed to sirolimus’s immunosuppressive property.

Patients with well-defined conditions stemming from mTOR overactivation, such as tuberous sclerosis (TSC) and lymphangioleiomyomatosis (LAM), present more suitable study subjects due to their targeted treatment needs and longer life expectancy. Both conditions involve mTOR pathway overactivation, with TSC primarily affecting pre- and post-natal and adolescent periods, and LAM affecting adult females [12]. Sirolimus’s therapeutic efficacy in these diseases has been well-documented [13–16] and fewer drugs are likely to interact with sirolimus due to these patients’ typically simpler medication histories.

This study leverages data from TSC and/or LAM patients treated with sirolimus for durations ranging from three months to ten years. By analyzing this data, we aim to elucidate the long-term safety profile of sirolimus and its potential effects on physical growth in pediatric populations.

## Methods

### Study design and participants

This retrospective analysis employed data from two nationwide, prospective cohort studies registered under ChiCTR-OOB-15,006,535 and NCT03193892. Informed consent was obtained from all participants (adults) or their legal guardians (pediatric patients). The study adhered to the Practical Clinical Practice (PCT) principles, the Declaration of Helsinki, and all relevant local regulations. Data collection spanned from September 30, 2010, to January 10, 2021.

**Fig. 1:**
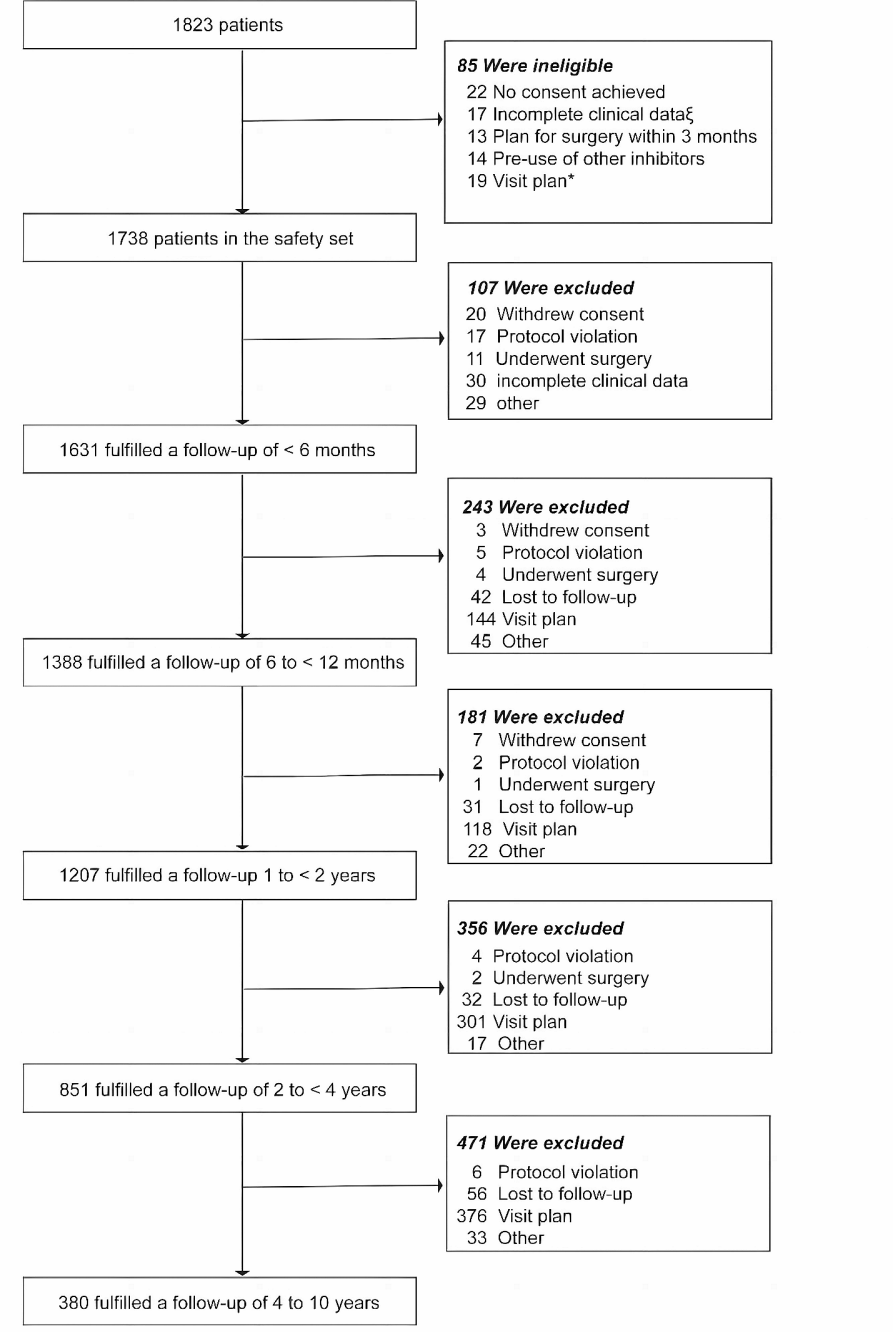
Study design. * The data collection is still ongoing. The date of the first visit in some patients made them fail to participate in a visit on January 20, 2021. ξ If no data can be used in the safety set, the piece of observation will be deleted

To represent real-world scenarios and enhance generalizability, we included patients diagnosed with TSC or LAM who had not previously received mTOR inhibitors. Individuals discontinuing sirolimus due to surgery, other interventions, or economic constraints were excluded during follow-up (as illustrated in Fig. 1).

We evaluated 16 primary outcome variables: physical growth (Z-scores for height, weight, and BMI), hematopoietic function (red blood cells, white blood cells, platelets, hemoglobin), liver function (alanine aminotransferase, aspartate aminotransferase), renal function (serum creatinine, uric acid, blood urea nitrogen), and blood lipid levels (total cholesterol, high-density lipoprotein, low-density lipoprotein, triglycerides). For height and weight, the changes were not specifically analyzed in female adult patients. These measurements were only used to determine whether there were any changes in their BMI levels. Additionally, we included descriptive analysis of adverse events reported by participants, categorized using the Medical Dictionary for Regulatory Activities (MedDRA) version 18.1 and graded by the National Cancer Institute Common Terminology Criteria for Adverse Events (CTCAE v4.03).

Participants received sirolimus via oral solution or tablet at a starting dosage of 1 mg/m^2^/day, with a maximum dose of 2 mg. Dose adjustments were made by a 2-week titration period to attain a trough blood concentration of 5 to 10 ng/ml. Subsequent fine-tuning, limited to increments or decrements no greater than 0.3 ml, was implemented to maintain concentrations within the target range. No further intervention was undertaken for concentrations persisting outside the target range after fine-tuning.

### Statistical analysis

Statistical analyses were performed using SAS software (version 9.4) and Python (version 3.9). Demographic characteristics are presented as mean ± standard deviation (SD) (normal distribution) or median (interquartile range (IQR)) (non-normal distribution). Locally weighted scatterplot smoothing (LOWESS) was used to generate trend curves for continuous variables.

For physical development parameters, we compared individual values against normative growth patterns of age-matched peers and categorized them as normal, lower than 2 SD, or higher than 2 SD. Stepwise regression analysis explored the influence of medication dosage, blood concentration, duration of medication use, and demographic data (adult/pediatric, sex, age, and concurrent medication use) on growth patterns.

Reference ranges for the variables varied across age groups. Therefore, for regression analyses of the variables related to hematopoietic function, liver function, renal function, and blood lipid levels, the numbers were categorized before entering the stepwise regression model. The reference intervals are shown in Table S1.

Statistical significance was set at *P* < 0.05, with two-tailed testing. In regression analyses, an odds ratio (OR) value is considered to have a strong effect if greater than 2 or less than 0.5.

## Results

A total of 1783 patients—1521 children and 217 adult females—ranging in age from 5 days to 69 years were included. During the follow-up period, 5078 observations of 1631 patients were gathered. Figure 1 delineates the distribution of patient follow-ups, and Table 1 presents baseline characteristics of the cohort. The number of patients in each follow-up is listed in Fig. 1. Table 1 lists the patient’s baseline characteristics. For males, the blood drug concentration was 5.86 ± 2.90 ng/ml, meanwhile for females, it was 6.14 ± 3.44 ng/ml. The median follow-up duration was 25 months (IQR: 12–43 months), with 380 individuals followed for over 4 years, 21 for over 8 years, and 4 reaching a 10-year follow-up.

**Table 1:** Characteristics of the participants at baseline

	Pediatric patients(*n* = 1521)	Adult patients(*n* = 217)
**Age(years)**	2.0 (0.8–5.6)	37.8 (31.4–45.6)
**Female, %**	654 (43.0%)	217 (100%)
**Height(cm)**	86 (73–112)	160 (158–163)
**Weight(kg)**	13 (10–21)	54 (7.7)
**BMI**	16.9 (15.6–18.8)	20.8 (19.4–23.0)
**Dose of sirolimus(mg)**	0.6 (0.4–0.8)	1 (1–2)
**Follow up (month)**	25 (11–44)	24 (12–41)
**RBC (10** ^**12**^ **/L)**	4.55 (4.26–4.83)	4.72 (4.42–5.11)
**Male**	4.58 (4.33–4.87)	
**Female**	4.52 (4.20–4.76)	4.72 (4.42–5.11)
**WBC (10** ^**9**^ **/L)**	8.00 (6.53–9.86)	6.11 (5.20–7.49)
**PLT (10** ^**9**^ **/L)**	296 (239–361)	269 (226–323)
**Male**	293 (236–357)	
**Female**	301 (241–369)	269 (226–323)
**HGB (g/L)**	124 (116–131)	141 (128–151)
**Male**	124 (116–132)	
**Female**	123 (116–131)	141 (128–151)
**ALT (U/L)**	14 (10–22)	13 (10–18)
**AST (U/L)**	28 (22–35)	18 (16–22)
**BUN (mg/dL)**	3.70(1.46)	4.10 (3.42–4.87)
**SCR (µmol/L)**	28 (22–37)	61 (55–67)
**UA (µmol /L)**	241.0 (197.7-289.5)	268 (224–320)
**TCH (mmol/L)**	3.85 (3.40–4.39)	4.36 (3.80–4.94)
**HDL (mmol/L)**	1.31 (1.07–1.56)	1.19 (0.34)
**LDL (mmol/L)**	2.17 (1.75–2.57)	2.58 (2.12–3.01)
**TG (mmol/L)**	0.84 (0.64–1.11)	1.00 (0.69–1.50)

Data are mean (SD), median (IQR), n, or n (%). BMI = body mass index. RBC = red blood cell count. WBC = white blood cell count. PLT = platelet count. HGB = hemoglobin. ALT = alanine transaminase. AST = aspartate aminotransferase. BUN = blood urea nitrogen. SCR = serum creatinine. UA = uric acid. TCH = total cholesterol. HDL = high density lipoprotein cholesterol. LDL = low density lipoprotein cholesterol. TG = triglyceride.

Analysis of height, weight, and BMI using established normative growth curves revealed that pre- and post-medication values mostly fell within the normal range, with a tendency towards the higher 50th percentile. Regression analysis of category deviations, narrowed to the pediatric group, showed that the dose of sirolimus was the only factor significantly associated with all three variables with ORs more than 2 or less than 0.5 (Table 2).

**Table 2:** Results of regression analysis

Variables	event	Factors	OR	95% CI	*P*-value
Z scores of height	> 2SD (higher)	dosage	7.236	3.357–15.598	< 0.0001
	<-2SD (lower)	dosage	0.061	0.011–0.346	0.0016
Z scores of weight	> 2SD (higher)	dosage	17.113	7.681–38.125	< 0.0001
	<-2SD (lower)	dosage	0.008	0.001–0.057	< 0.0001
Z scores of BMI	> 2SD (higher)	dosage	30.239	12.082–75.681	< 0.0001
	<-2SD (lower)	dosage	0.054	0.015–0.193	< 0.0001
		adult	0.129	0.042–0.394	0.0003
RBC	higher	female	5.115	3.444–7.596	< 0.0001
	lower	female	0.248	0.110–0.560	0.0008
WBC	higher	dosage	0.321	0.228–0.451	< 0.0001
	lower	-	-	-	-
PLT	higher	-	-	-	-
	lower	adult	62.791	9.900-398.254	< 0.0001
HGB	higher	female	161.786	40.043-653.676	< 0.0001
		adult	2.713	1.404–5.241	0.0030
	lower	adult	9.530	2.970–30.582	0.0002
		female	0.400	0.312–0.511	< 0.0001
		dosage	0.221	0.132–0.368	< 0.0001
ALT	higher	adult	0.114	0.036–0.364	0.0002
AST	higher	dosage	0.466	0.302–0.721	0.0006
SCR	higher	-	-	-	-
UA	higher	adult	5.046	2.604–9.778	< 0.0001
BUN	higher	-	-	-	-
TCH	higher	adult	0.492	0.367–0.659	< 0.0001
HDL	lower	dosage	2.452	1.712–3.513	< 0.0001
LDL	higher	dosage	0.203	0.146–0.282	< 0.0001
TG	higher	adult	0.338	0.246–0.464	< 0.0001

The table only displays results with a strong effect, where the OR values are greater than 2 or less than 0.5. The complete results of the regression analysis are in Supplementary Table 2. For some variables, no factors with a strong effect; therefore, only the names of these variables are listed

**Fig. 2:**
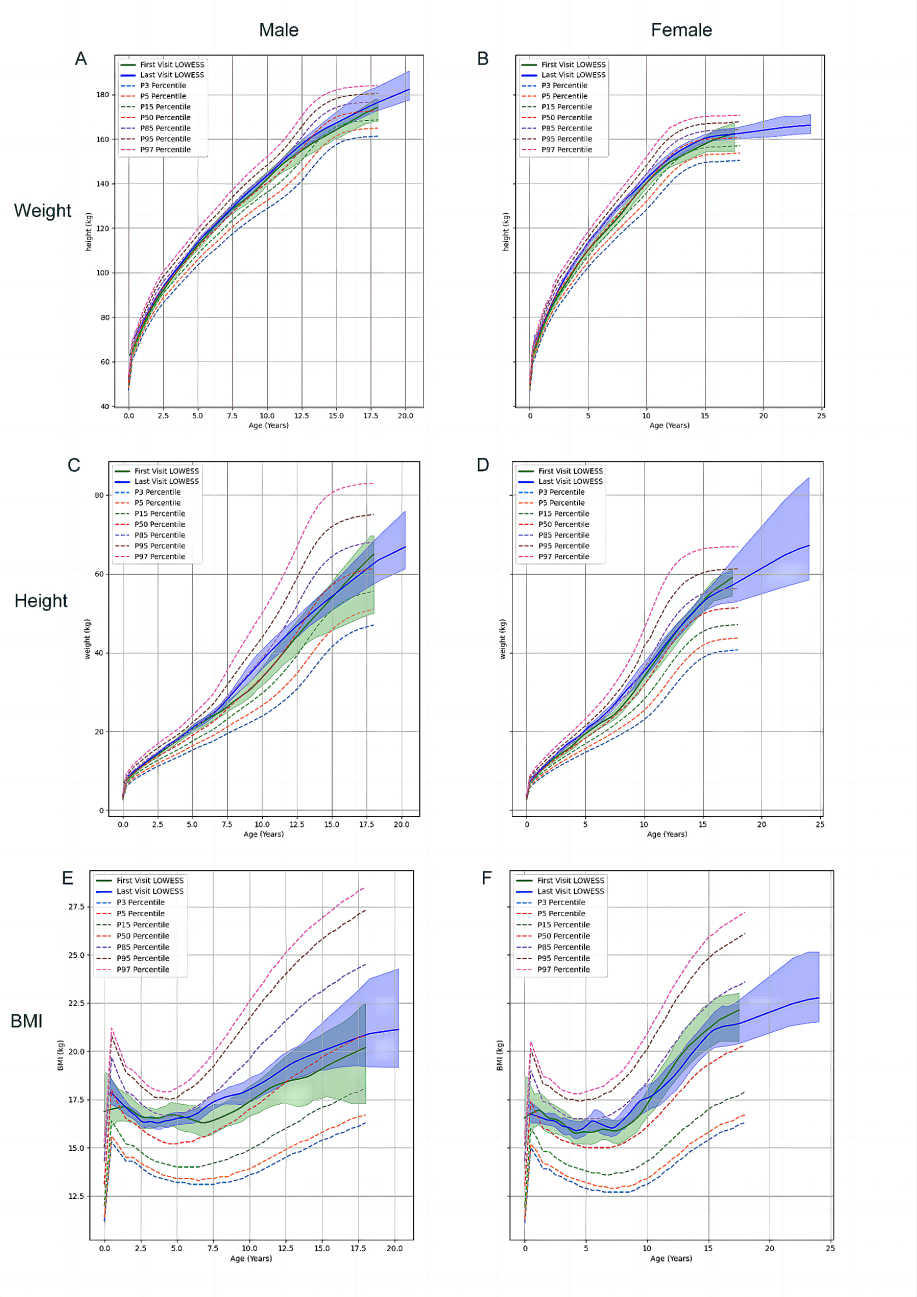
Mapping distribution of patients’ height, weight, and BMI among peers at first and last follow-up. The shaded area around the distribution fitting curve represents the 95% CI. The dashed lines from top to bottom indicate different percentiles of growth levels (97%, 95%, 85%, 50%, 15%, 5%, 3%). The green solid line represents the curve at the first visit, and the bluish-purple solid line represents the curve at the last visit

Transient increases in RBC, WBC, HGB, and PLT levels were noted within the first year of follow-up, subsequently returning to baseline values (Fig. 2). Regression analysis identified gender as a major predictor; females had a much-increased chance of increasing RBC levels (OR: 5.12). The gender gap was even more significant for HGB levels, with females having an OR of 161.79 for elevated levels. For both HGB and WBC levels, there was a negative correlation between sirolimus dosage and these variables (Table 2). Other variables had modest influence (Table S2).

In terms of liver function, children and adult females displayed distinct patterns. Adult females showed no significant changes in ALT levels, while pediatric patients experienced a transient decrease in early follow-up and then maintained those levels. AST levels remained stable in adults, while pediatric values gradually decreased. Regression analysis indicated that this decrease was negatively correlated with the dose of sirolimus (OR: 0.47) (Table 2).

**Fig. 3:**
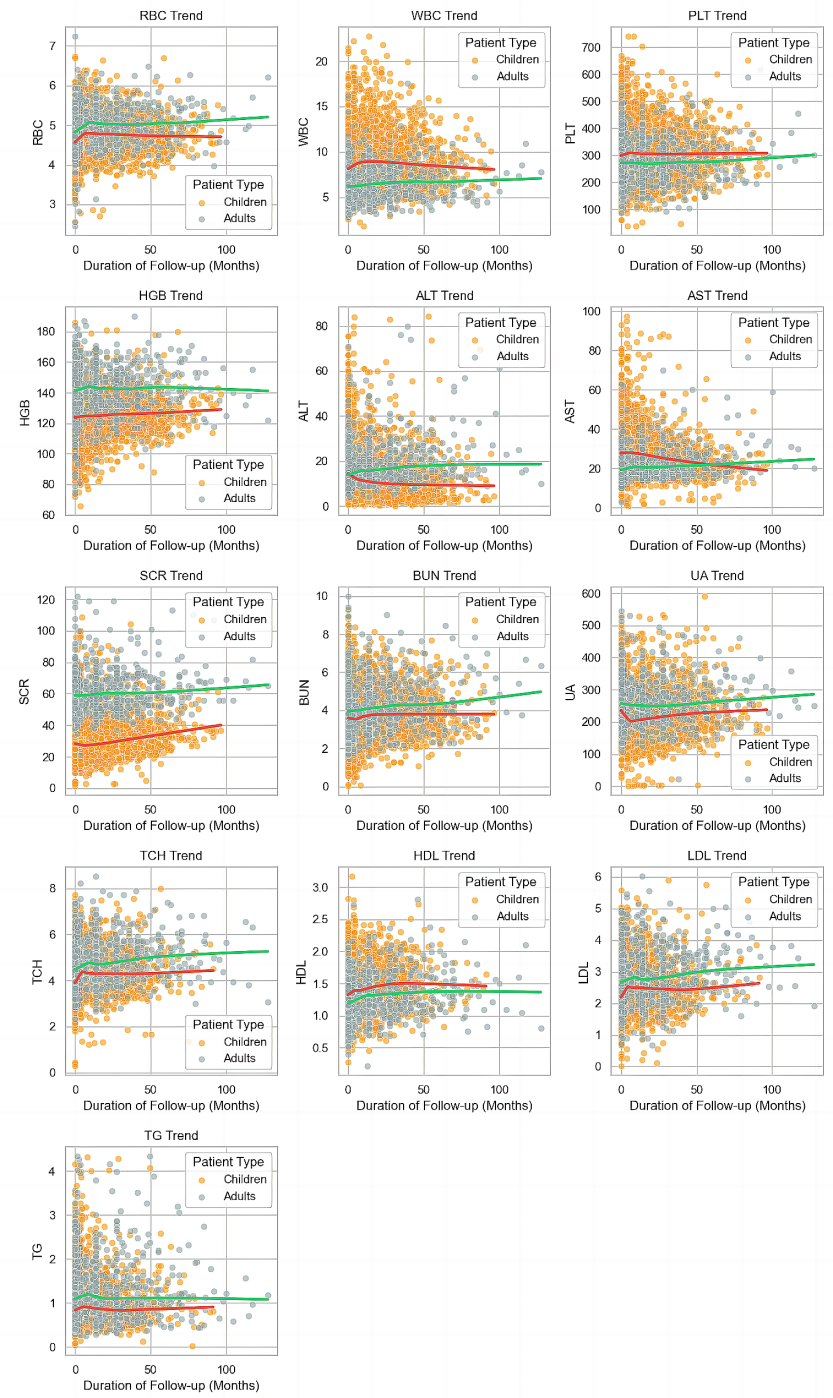
Changes in 13 safety-related indicators throughout the follow-up period. The green line represents the fitting curve for adults, while the red line represents the fitting curve for children. The gray scatter points represent the specific value distribution for adult females, and the orange scatter points represent the specific value distribution for children

Adults had greater numerical values for SCR, BUN, and UA than children (Fig. 3). Except for a temporary drop in UA in children during early follow-up (within a year), changes were minimal, with only minor increases. After categorizing the data, regression analysis revealed no significant association between sirolimus use and any renal parameter, but adult females had a larger proportion of increased UA compared to children (Table 2).

The primary rise in TCH in both children and adults occurred early in medication usage (less than one year), with adults having higher levels than children. Similar patterns were identified in LDL changes (Fig. 3). Changes in HDL were small, and it was the only lipid indication in which children had higher levels than adults. Triglyceride levels did not vary much throughout long-term follow-up. The dose of sirolimus demonstrated a significant correlation with HDL and LDL, but not with TG or TCH, with the latter two generally displaying a higher level in adult females than children (Table 2).

Adverse events analysis revealed stomatitis as the most frequent condition (920/1738 cases, 52.9%), and it usually resolved within a week. The management of AEs is listed in Figure S1. Gastrointestinal symptoms were also common, including diarrhea, nausea, abdominal pain, reduced appetite, vomiting, and distention (Table 3). Menstrual irregularities affected 48.5% of adult females (112 of 217), with durations ranging from 6 to 24 months, and the longest case lasting 80 months. Serious adverse events were infrequent, with two cases of grade III or higher identified during the research. One patient experienced lower limb edema and urinary dysfunction after three months on medication but fully recovered after hospital treatment, successfully resuming sirolimus three months later without recurrence of similar AEs. Another patient, however, died from an unknown cause after two years of therapy. Medication discontinuation was observed in 38 participants at different stages of follow-up, with adverse effects directly prompting cessation in seven instances: one due to recurrent stomatitis and six due to recurrent infections. Economic constraints, prognostic concerns, and therapeutic adjustments were the primary reasons for treatment discontinuation in 15, 10, and 6 patients, respectively (Table 3).

**Table 3:** The summary of descriptive adverse events in all the patients throughout the follow-up

	Pediatric patients		Adult patients		All patients¶	
AEs	Percentage in patients (*N* = 1521)	Percentage in all follow-up (*N* = 4408)	Percentage in patients (*N* = 217)	Percentage in all follow-up (*n* = 670)	Percentage in patients (*N* = 1738)	Percentage in all follow-up (*n* = 5078)
Diarrhea	80 (5·3%)	93 (2·1%)	11 (5·1%)	14 (2·1%)	91(5·2%)	107 (2·1%)
Nausea	7 (0·46%)	12 (0·3%)	1 (0·46%)	1 (0·15%)	8 (0·46%)	13 (0·26%)
Abdominal pain	37 (2·4%)	40 (0·9%)	3 (1·4%)	3 (0·45%)	40 (2·3%)	43 (0·85%)
Decreased appetite	40 (2·6%)	76 (1·7%)	3 (1·4%)	5 (0·75%)	43 (2·5%)	81 (1·6%)
Vomiting	3 (0·20%)	3 (0·07%)	0	0	3 (0·17%)	3 (0·06%)
Distention	15 (1·0%)	21 (0·48%)	3 (1·4%)	3 (0·45%)	18 (1·0%)	25 (0·49%)
Upper respiratory tract infection ‡	113 (7·4%)	152 (3·4%)	2 (0·92%)	2 (0·30%)	115 (6·6%)	154 (3·5%)
Menstruation abnormal⁎	12 (0·79%)	15 (0·34%)	112 (48·5%)	194 (28·9%)	124 (7·1%)	209 (4·8%)
Rash acneiform†	61 (4·0%)	89 (2·0%)	113 (52·1%)	186 (27·8%)	174 (10·0%)	275 (5·4%)
Alopecia	0	0	1 (0·46%)	3 (0·45%)	1 (0·06%)	3 (0·06%)
Edema of lower extremities	2 (0·13%)	2 (0·05%)	2 (0·92%)	2 (0·30%)	4 (0·23%)	4 (0·08%)
Adynamia	1 (0·07%)	1 (0·02%)	2 (0·92%)	2 (0·30%)	3 (0·17%)	3 (0·06%)
Eyelid edema	0	0	1 (0·46%)	1 (0·15%)	1 (0·06%)	1(0·02%)
Dizziness	1 (0·07%)	1 (0·02%)	1 (0·46%)	1 (0·15%)	2 (0·12%)	2 (0·04%)
Pruritus	0	0	2 (0·92%)	2 (0·30%)	2 (0·12%)	2 (0·04%)
Back pain	0	0	1 (0·46%)	1 (0·15%)	1 (0·06%)	1(0·02%)
Ulcerative stomatitis	789 (51·9%)	1229 (27·9%)	131 (56·7%)	227 (34·7%)	920 (52·9%)	1456 (28·7%)
Urinary incontinence	1 (0·07%)	2 (0·05%)	0	0	1 (0·06%)	2 (0·04%)
Headache	31 (2·0%)	41 (0·91%)	5 (2·3%)	7 (1·0%)	36 (2·1%)	18 (9·5%)
Somnolence	2 (0·13%)	2 (0·05%)	0	0	2 (0·12%)	2 (0·04%)
Gum hyperplasia	0	0	1 (0·46%)	2 (0·30%)	1 (0·06%)	2 (0·04%)
Arthromyalgia	1 (0·07%)	1 (0·02%)	2 (0·92%)	3 (0·45%)	3 (0·17%)	4 (0·08%)
Encephalitis	1 (0·07%)	1 (0·02%)	0	0	1 (0·06%)	1(0·02%)
Death	1 (0·07%)	1 (0·02%)	0	0	1 (0·06%)	1(0·02%)
No AEs	**566(37·2%)**	**2975 (67·5%)**	**36(16·5%)**	**231 (35·5%)**	**602 (34·6%)**	**1871 (63·1%)**
AEs of Grade 3 and above	**2 (0·13%)**	**2 (0·05%)**	**0**	**0**	**2 (0·12%)**	**2 (0·04%)**

Data are *n*, %, the adverse events are coded using Medical Dictionary for Regulatory Activities (MedDRA) V.18.1

⁑If the patient has upper respiratory tract infection and cough at the same time, the adverse event currently is recorded as an upper respiratory tract infection

†Because patients with tuberous sclerosis, especially those with childhood diseases, are more likely to have other rash-like lesions such as facial angiofibroma, it is sometimes difficult to assess whether the rash appears after taking sirolimus. Therefore, we only recorded rash-like lesions in pediatric patients as adverse events when the rash increased significantly after taking the drug, or when there were rashes in other areas or with features different from tuberous sclerosis

⁎Only adult female patients were included when calculating the incidence rate of irregular menstruation

¶ The total number here is the total number of patients in the safety data set

## Discussion

This study obtained subjects from a population of all ages and is designed to give a panoramic view on whether sirolimus is ready to expanding its indications. Patients in this study aged from 5 days to 69 years, and 380 patients underwent a follow-up of 4 to 10 years. Also, 16 dependent variables in the study can reflect the impact of sirolimus on physical growth, hematopoietic function, liver function, renal function, and lipid profiles. Other descriptive AEs were also recorded and analyzed. Besides, patients in the study were with a simple drug-use history and better health conditions, which reduced the possibility of wrongly attributing unrelated symptoms to sirolimus medication.

This careful curation of the study population enhances the reliability of our findings regarding the medication’s effects and tolerability.

Sirolimus does not negatively affect the physical growth of pediatric patients. The growth and development fitting curves before and after medication use showed similar patterns. However, intriguing findings emerged when examining individuals with pre-existing abnormal height, weight, or BMI. Stepwise regression analysis identified sirolimus dosage as the sole factor demonstrating positive correlations with values exceeding the normal range and negative correlations with values below the normal range, with both correlations exhibiting strong effects. This finding suggests a potential dose-dependent effect of sirolimus on growth patterns in children. This study employed stepwise regression analysis, incorporating variables into the model only when they increased overall accuracy, thus avoiding the potential for misinterpretation due to the intrinsic correlations among multiple factors. The study also thoroughly considered the impact of demographic information such as age and gender and included drug concentration and duration of use. Also, the analysis excluded adult females to avoid potential confounding factors. On this basis, the correlation between dosage and growth development is credible. These results are different from some previous studies reporting negative growth impacts associated with sirolimus use in renal transplant patients [17, 18]. Neither lower growth velocity nor smaller changes in height was observed in our study.

This discrepancy underscores the potential influence of pre-existing health conditions and the dosage of sirolimus. Renal transplant patients are usually subjected to transplantation due to end-stage renal disease, which can potentially affect the growth pattern of patients [19]. This situation can lead to metabolic disorders, malnutrition, and endocrine dysfunction, all of which can negatively impact growth and development. Post-transplantation, renal transplant patients generally require long-term use of immunosuppressants and other medications to prevent rejection and manage complications. These medications, such as corticosteroids, are known to have adverse effects on growth in children and adolescents [20]. Additionally, the median dose of sirolimus in our study (0.6 mg) was lower compared to other studies (2-5 mg/day) [21].

The influence on growth also cannot be fully explained by controlling the progression of the primary disease (TSC), as neither the blood concentration of sirolimus nor the duration of its use was related to this outcome. More studies are needed to elucidate the underlying mechanisms.

The effect of sirolimus on hematopoiesis is different from the results of the previous studies [7, 22, 23]. AEs in the previous studies included mild and reversible thrombocytopenia, anemia, and leukopenia [24]. We did not observe any of the above. In fact, the impact of sirolimus use on hematopoietic function is minimal. Even after completing regression analysis, only WBC and HGB levels showed a certain degree of association with the drug’s dosage. Interestingly, the dosage is negatively correlated with WBC levels being above normal value and negatively correlated with HGB levels being below the normal value. In previous study, for different murine models, inhibition of mTORC1 can play opposing roles in the maintenance of hematopoietic stem cells (HSC) [25, 26]. HSC self-renewal and lineage commitment depend on complex interactions with the microenvironment [27], and thus, the different results may be related to many other factors including their drug use profiles and patients’ conditions. For example, in renal transplant patients, a combination of functional iron deficiency and sirolimus use can lead to anemia [28], but this phenomenon was not frequently observed in other studies [14, 29].

We did not observe sirolimus-associated hepatotoxicity. Since children and adults have different reference ranges for ALT, although the ALT levels in adult females were numerically higher than in adults, the proportion of abnormalities was actually lower than in children, with the OR value for adults compared to children being 0.11. However, for AST abnormalities, the dosage of sirolimus showed a negative correlation. This to some extent demonstrates that sirolimus does not possess hepatotoxicity. Hepatic metabolism, particularly the cytochrome P450 system, plays a significant role in the metabolism of sirolimus [30]. Hepatotoxicity is rarely reported and is mostly seen in patients post-transplantation [31], suggesting it may be related to certain diagnostic or therapeutic factors but not a primary concern for all patients.

Sirolimus has no evident influence on renal function. The results of the regression analysis show that changes in SCR, BUN, and UA overall are not related to the use of sirolimus, although these indicators showed a mild increase over time with follow-up. The reference intervals for different ages vary, and typically, with aging, these values also increase [32]. However, a decrease in the value of UA was observed within a 1-year follow-up in pediatric patients. Coupled with the relatively small increase in SCR and BUN as patients age, it can at least be said that the long-term use of sirolimus did not negatively impact renal function.

The influence of sirolimus on HDL, LDL, and TG was different. An obvious increase in all variables mainly occurred in short term, but then only the level of TCH and LDL increased slightly, and as for HDL and TG, their level remained stable in a long term. For health population, apart from HDL, the value of the other three variables is positively related to age [33], so the increase in the long-term follow-up may not be caused by sirolimus exclusively.

The descriptive AEs are mild in general. Stomatitis and gastrointestinal complications are the most common but could resolve spontaneously or by symptomatic treatment or reducing the dose of sirolimus. Two patients encountered grade III or above AEs. One developed lower limb edema and urinary dysfunction at 3-month medication. Edema has been reported in transplant recipients, and a previous study proved that mTOR inhibition may cause edema [34]. One patient died after using sirolimus for two years, but his caregivers did not report the cause. Another common adverse event is ovarian toxicity. Menstrual disorders were observed in 48.5% (112/217), and the longest lasted for 80 months. One RCT study proved that low-dose oral sirolimus increased the risk of menstrual-cycle disturbances and ovarian cysts [35]. Thus, monitoring sirolimus-associated ovarian toxicity in female patients of childbearing age is recommended.

In this study, we noted that although the dosage of sirolimus, blood drug concentration, duration of medication use, and concurrent use of other medications were included in the study, only the dosage was strongly associated with abnormal values. We observed that within a relatively lower dosage range, more sirolimus seemed to be associated with more beneficial outcomes, whether in terms of growth patterns, WBC and HGB levels, or changes in lipid levels. Our findings suggest that the effects of sirolimus may exhibit a certain dose-response reversal effect. That is to say, below a specific dosage, its advantages increase with the increase in dosage, but these results will be reversed when the dosage is larger than a threshold. These are preliminary results of our study, and further exploration is ongoing.

At present, the potentials of sirolimus are continually being explored. This study evaluated sirolimus’s safety profile in all-age groups. Although there is no control group, this study has set up the safety profile of sirolimus in actual clinical scenarios by analyzing changes of 16 numerical variables and a descriptive variable. This study provides a basis for using sirolimus to treat other mTOR-related diseases. Those who are committed to expanding indications of sirolimus can gain courage and confidence from our results.

## Conclusions

Sirolimus’s long-term administration is not associated with adverse effects on children’s physical growth, nor significant alterations in hematopoietic, liver, renal function, or lipid levels. A potential positive dose-dependent influence on growth merits further exploration. Stomatitis and gastrointestinal complications are the most common AEs and ovarian toxicity should also be taken into consideration.

### Electronic supplementary material

Below is the link to the electronic supplementary material.

Supplementary Material 1

## Data Availability

Deidentified individual participant data (including data dictionaries) will be made available, in addition to study protocols, the statistical analysis plan, and the informed consent form. The data will be made available upon publication to researchers who provide a methodologically sound proposal for use in achieving the goals of the approved proposal. Proposals should be submitted to *zouliping21@hotmail.com*.

## Abbreviations

mTOR: mechanistic (mammalian) target of rapamycin
mTORC1: mechanistic (mammalian) target of rapamycin complex 1
TSC: Tuberous sclerosis complex
LAM: Lymphangioleiomyomatosis
BMI: Body mass index
CBC: Complete blood count
RBC: Red blood cell count
WBC: White blood cell count
PLT: Platelet count
HGB: Hemoglobin
ALT: Alanine transaminase
AST: Aspartate aminotransferase
BUN: Blood urea nitrogen
SCR: Serum creatinine
UA: Uric acid
TCH: Total cholesterol
HDL: High density lipoprotein cholesterol
LDL: Low density lipoprotein cholesterol
TG: Triglyceride
AEs: Adverse events
