# Supplementary material for: Long-term safety and influence on growth in patients receiving sirolimus: a pooled analysis
Source: Orphanet J Rare Dis. 2024 Aug 15;19:299. doi: 10.1186/s13023-024-03243-5 (PMC11325711; doi:10.1186/s13023-024-03243-5)
Supplement: Supplementary file 1 — Supplementary Material 1 [file 13023_2024_3243_MOESM1_ESM.pdf]

Table S1 The reference range for outcome variables

	Sex	age (Years)	lower reference	upper reference
RBC	Male	ALL	4.08	5.8
	Female	ALL	3.7	5.2
HGB	Male	ALL	121	176
	Female	ALL	113	157
PLT	ALL	ALL	132	353
WBC	ALL	< 0.5	3.48	20
	ALL	0.5-2	3.48	12
	ALL	>2	3.48	9.5
ALT (U/L)	ALL	< 1		45.3
	ALL	1		39.7
	ALL	2		28.3
	ALL	3		24.4
	ALL	4		23.6
	ALL	5-9		20.9
	Male	10-11		30.2
	Female			24
	Male	12-13		30.8
	Female			30.7
	Male	14-17		39.3
	Female			30.5
	Male	Adult		50
	Female	Adult		37
AST (U/L)	ALL	< 1		49
	ALL	1		49
	ALL	2		49
	ALL	3		44
	ALL	4		44
	ALL	5-9		40
	Male	10-11		39
	Female			35
	Male	12-13		39
	Female			35
	Male	14-17		36
	Female			26
	Male	Adult		40
	Female	Adult		35
CRE (μmol/L)	ALL	< 1		36.29
	ALL	1		38.74
	ALL	2		42.56
	ALL	3		44.06
	ALL	4		46.57

	ALL		5		50.12
	ALL		6		54.13
	ALL		7		55.36
	ALL		8		57.3
	ALL		9		60.38
	Male	10-11			68.81
	Female	10-11			64.08
	Male	12-13			98.25
	Female	12-13			76.49
	Male	14-17			105.13
	Female	14-17			105.13
BUN (mmol/L)	ALL	ALL			5.41
UA (μmol/L)	ALL	< 1			398
	ALL	1~4			376.76
	ALL	5~9			408.92
	Male	10~11			491
	Female	10~11			455
	Male	12~13			617
	Female	12~13			473
	Male	14~17			606
	Female	14~17			437
	Male	Adult			416
	Female	Adult			357
TCH	ALL	<6			4.56
	Male	<9			4.9
	Female	<9			4.9
	Male	< 12			4.85
	Female	< 12			4.85
	Male	< 15			4.56
	Female	< 15			4.72
	Male	< 18			4.8
	Female	< 18			4.93
	ALL	Adult			5.71
HDL	ALL	<6		0.85	
	Male	<9		1.13	
	Female	<9		1.13	
	Male	< 12		1.08	
	Female	< 12		1.08	
	Male	< 15		0.94	
	Female	< 15		1.04	
	Male	< 18		0.85	
	Female	< 18		0.96	
	ALL	Adult		0.91	
LDL	ALL	<6			1.79

	Male	<9	3.19
	Female	<9	3.19
	Male	< 12	3.14
	Female	< 12	3.14
	Male	< 15	2.85
	Female	< 15	2.98
	Male	< 18	2.81
	Female	< 18	2.81
	ALL	Adult	3.37
TG	ALL	<9	0.88
	Male	< 12	1.23
	Female	< 12	1.23
	Male	< 15	1.32
	Female	< 15	1.25
	Male	< 18	2
	Female	< 18	2
	ALL	Adult	1.7

Table S2 All variables with statistical significance in regression analysis

Variables	event	Factors	OR	95% CI	P-value
Z scores of height	> 2(higher)	age	1.000	0.999-1.000	<.0001
		dose	7.236	3.357-15.598	<.0001
	<-2 (lower)	age	1.001	1.000-1.001	0.0033
		dose	0.061	0.011-0.346	0.0016
Z scores of weight	> 2(higher)	age	0.999	0.999-1.000	<.0001
		dose	17.113	7.681-38.125	<.0001
		female	0.664	0.484-0.911	0.0112
	<-2 (lower)	age	1.001	1.001-1.001	<.0001
		dose	0.008	0.001-0.057	<.0001
Z scores of BMI	> 2(higher)	dose	30.239	12.082-75.681	<.0001
		age	0.999	0.999-1.000	<.0001
		adult	0.135	0.058-0.315	<.0001
		duration	0.987	0.976-0.998	0.0254
		female	0.6	0.419-0.86	0.0054
		ASMs⁑	0.814	0.696-0.952	0.0099
	<-2 (lower)	age	1.001	1.000-1.001	0.0001
		dose	0.054	0.015-0.193	<.0001
		adult	0.129	0.042-0.394	0.0003
		ASMs	1.196	1.014-1.409	0.0332
RBC	higher	age	1.044	1.022-1.608	<.0001
		duration	1.007	1.000- 1.014	0.0364
		concentration	1.107	1.070-1.145	<.0001
		female	5.115	3.444-7.596	<.0001
		ASMs	0.654	0.554-0.773	<.0001
		adult	1.830	1.334-2.511	0.0002
	lower	female	0.248	0.110-0.560	0.0008
		ASMs	1.607	1.268-2.036	<.0001

WBC	higher	age	1.000	1.000-1.000	<.0001
		dose	0.321	0.228-0.451	<.0001
		duration	1.027	1.021-1.033	<.0001
	lower	age	1.000	1.000-1.000	0.0098
PLT	higher	age	1.000	1.000-1.000	<.0001
		ASMs	0.815	0.748-0.889	<.0001
		dose	0.640	0.473-0.865	0.0037
	lower	age	1.000	1.000-1.000	0.0006
		adult	62.791	9.900-398.254	<.0001
HGB	higher	age	1.000	1.000-1.000	0.0078
		female	161.786	40.043-653.676	<.0001
		dose	1.745	1.262-2.413	0.0008
		adult	2.713	1.404- 5.241	0.0030
	lower	age	1.000	1.000-1.000	0.0157
		adult	9.530	2.970 - 30.582	0.0002
		concentration	1.071	1.037-1.0107	<.0001
		female	0.400	0.312-0.511	<.0001
		dose	0.221	0.132-0.368	<.0001
		ASMs	1.124	1.021- 1.237	0.0171
ALT	higher	age	1.000	1.000-1.000	0.0079
		adult	0.114	0.036-0.364	0.0002
		concentration	1.087	1.042 - 1.133	<.0001
		duration	1.019	1.019 -1.028	<.0001
		ASMs	0.601	0.497-0.728	<.0001
AST	higher	dose	0.466	0.302-0.721	0.0006
		concentration	1.165	1.121-1.211	<.0001
		ASMs	0.546	0.441-0.675	<.0001
SCR	higher	-	-	-	-
UA	higher	age	1.000	1.000-1.000	<.0001

		adult	5.046	2.604-9.778	<.0001
		concentration	0.904	0.856-0.955	0.0003
		duration	1.013	1.006-1.021	0.0002
		female	0.672	0.478-0.945	0.0224
		ASMs	1.204	1.052-1.377	0.0068
BUN	higher	age	1.000	1.000-1.000	<.0001
		dose	1.543	1.098-2.168	0.0124
		concentration	0.888	0.827-0.942	0.0010
		duration	1.012	1.005-1.020	0.0015
		female	0.603	0.424-0.860	0.0052
		ASMs	1.218	1.061-1.398	0.0051
TCH	higher	dose	0.611	0.455-0.821	0.0011
		adult	0.492	0.367-0.659	<.0001
HDL	lower	dose	2.452	1.712-3.513	<.0001
		duration	0.983	0.969-0.997	0.0162
		female	1.904	1.158-3.132	0.0112
LDL	higher	age	1.000	1.000-1.000	<.0001
		dose	0.203	0.146-0.282	<.0001
		duration	1.013	1.005-1.021	0.0014
		concentration	1.067	1.023-1.113	0.0027
TG	higher	concentration	0.892	1.064-1.145	<.0001
		female	1.67	1.295-2.152	0.0003
		dose	0.554	0.417-0.737	<.0001
		adult	0.338	0.246-0.464	<.0001

ASMs⁑ Anti-seizure Medications

Figure S1 The management of the adverse events

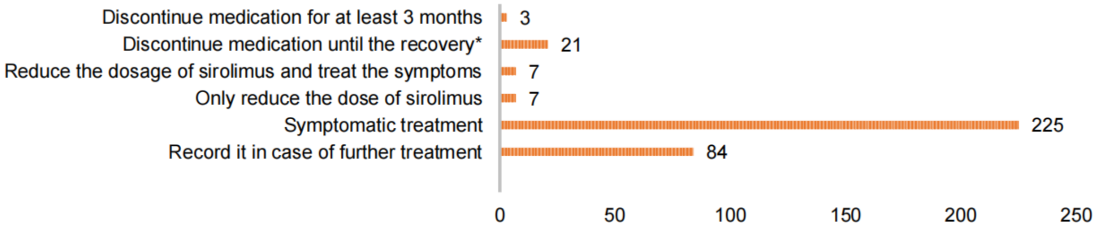

*The drug use would be resumed after the adverse event recovered, and the period was less than 3 months. A patient may have multiple adverse events and be given multiple treatments. What was registered in this figure was the highest level of treatment. For example, if a patient was treated symptomatically for stomatitis and his acne rash was only recorded but was not treated, then the management of adverse events was recorded as “symptomatic treatment”. In this way, this map can reflect the highest level of treatment for each patient.
